# Pulling the purse strings: Are there sectoral differences in political preferencing of Chinese aid to Africa?

**DOI:** 10.1371/journal.pone.0232126

**Published:** 2020-04-22

**Authors:** Carrie B. Dolan, Kaci Kennedy McDade

**Affiliations:** 1 Department of Health Sciences, William and Mary, Williamsburg, Virginia, United States of America; 2 Duke Global Health Institute, Duke University, Durham, North Carolina, United States of America; University of Glasgow, UNITED KINGDOM

## Abstract

**Objective:**

China is emerging as an increasingly important player in the global development space, but may be less bound to compacts that aim to curb political preferencing and therefore may produce less yield in terms of impact toward Sustainable Development Goals. This research tests the hypothesis that the disproportionate aid allocation to the birth regions of the current African political leaders that applies to some sectors more than others.

**Design:**

We applied a two-part model to first estimate the probability that a region receives an aid project. Then when at least one aid project is present in a leader’s birth region, we estimated the mean amount of aid the region received.

**Setting:**

This analysis covers 699 subnational units (first administrative level) across 44 African countries over 2000–2014. These administrative units were compiled into a region year panel resulting in 10,485 observations.

**Results:**

Birth regions of the current political leader are significantly more likely than the average of all of the regions to receive education (1.3 percentage points), social infrastructure and services (1.2 percentage points), and energy aid (1.7 percentage points). No significant association was found between aid flows to the birth region of the current political leader in the agriculture, communication, education, government, health or transportation sectors. Within the education sector, the coefficients for birth region are positive in both parts and statistically significant. Both the probability of aid allocation and the amount of aid conditional on any projects increase in the birth region of the current political leader.

**Conclusions:**

This sector-specific analysis provides a more nuanced picture of Chinese aid than previous analyses that determine the presence of political preference according to aggregate aid flows. The sectors where political preferencing exists are also those sectors that are typically associated with limited counterfactual-based program evaluation. We present evidence that demonstrates the importance of disaggregating aid flows in order to support a new policy framework designed to target the Sustainable Development Goals.

## 1. Introduction

Chinese aid, although not a new phenomenon, has dramatically expanded in recent years [1]. According to a recent estimate, from 2010 to 2015, emerging bilateral donors increased their annual aid by 47%; China is the biggest donor among them [2]. Many of these emerging donors, including China, are not part of the Organization for Economic Cooperation and Development’s (OECD) Development Assistance Committee (DAC), and therefore are not required to report their aid flows nor abide by conventional aid effectiveness principles [3].

China has published ad hoc documentation of its foreign aid contributions, yet its definition of aid does not align with OECD standards. Its self-published foreign aid flows are at the aggregate level and do not provide much insight into specific projects or recipients[1]. In response to this data transparency challenge, several estimates of Chinese aid were developed to attempt to align aid flows with OECD standards. Using common standards to measure and track aid also allows for comparison between OECD and non-OECD donors. Strange et al. estimated that China’s financial commitments to Africa 2000 to 2011 accounted for approximately $75 billion US dollars through 1673 projects [4]. Other analyses of Chinese aid efforts show that China’s net foreign aid was $4.5 billion dollars in 2011, $5.7 billion in 2012, and $7.1 billion in 2013 [5].

The lack of transparent aid information has led some to label China a ‘rogue donor’ who pursues aid for its own benefit and undermines development policy [6–8]. Recent empirical analyses show this characterization of China as a rogue donor paints an inaccurate picture [6,7]. Nonetheless, China’s approach to aid provision differs from that of OECD members and warrants deeper scrutiny.

China has largely portrayed its development philosophy as “win-win” through which China offers the provision of infrastructure in exchange for access to natural resources [9]. China aligns its development policies with its foreign policy principle of non-interference in the domestic politics of other countries [10]. This raises the question as to whether non-interference makes China a favorable, or even preferred, aid provider to its recipients and to what extent it poses risks such as supporting political favoritism and corruption while weakening accountability and/or respect for human rights. Dreher et al. found that Chinese aid leads to “political preferencing”. A dataset covering 1,650 Chinese development projects from 2000 to 2012 showed that when leaders hold power their birth regions receive substantially more Chinese aid flows than other subnational regions [11]. This suggests that Chinese aid flows are susceptible to manipulation, a phenomenon that may not occur if China was bound to the same transparency principles as OECD member countries. However, little is known about the determinants of aid flows at the sector level and consequently which sectors are most subject to manipulation. Both the inability to disaggregate aid information by sector as well as the absence of comprehensive data on Chinese aid flows has led to a small body of research aimed at understanding the determinants of China’s aid provision. Dreher and Fuchs determine that Chinese aid allocation is significantly influenced by both political and commercial interests [10]. Other evidence on determinants of bilateral aid from Mishra suggests that recipient need in the form of population, donor interests, and governance are important [12]. However, Chinese ODA allocation may rely on different motivations depending on the sector examined [7]. In recent years, there have been several studies that examined sectoral motivations for Chinese aid allocation. This literature remains thin and mostly focuses on the health sector. Guillion and Mathonnat studied the factors associated with Chinese health ODA to Africa in the 2006–2013 period. They find that Chinese health aid was responsive to economic needs (measured as Gross Domestic Product per capita) and favored countries with limited ability to finance health projects using national funds. There was no evidence of health aid allocation based on district level needs such as life expectancy, child and maternal mortality, or malaria prevalence [13]. Results do confirm the idea that health aid is part of China’s foreign policy as non-adherence to the one-China policy makes the receipt of Chinese health aid unlikely. In other work Zhao, Kennedy, and Tang find GDP as a significant determinant of Chinese health aid allocation[14]. Using cross-sectional data, Zhao et al. highlights good governance and human rights as motivations for allocation and finds no evidence of allocation based on bilateral trade or UN voting affinity[14].

The literature provides very little comprehensive evidence on the more nuanced determinants of Chinese aid allocation at sector levels. Guillon and Mathonnat refine the literature by studying the determinants of Chinese aid allocation across three broad sectors (social, economic, and production) over the 2000–2014 period[13]. Their results point to the influence of GDP in the social and production sectors, but not in the economic sectors[13]. They find that weaker governance matters in the provision of economic and production aid, but not in the social sector. When considering all three broad sectors together their results contradict previous aggregate level research, and indicates that African countries with high endowments of natural resources tend to receive more Chinese ODA[13]. In sum, the sparse literature concludes that there is a need to disaggregate the analysis by ODA sectors as analyzing overall aid to Africa can be misleading.

The current study examines political preferencing in China by aid sector, following other studies that take a sector specific approach to understanding aid allocation in China [14–16]. Aggregate analyses such as Dreher et al.’s, illuminated an alarming phenomenon[11]. However, such aggregate analyses ignore the fact that China’s aid system has traditionally been fragmented in both funding and in management, and therefore lacked a unified cross-sector strategy. Chinese aid was only recently reorganized under a single aid agency, the Chinese International Development and Cooperation Agency, and therefore analyses of aggregate flows may mask the behaviors and vulnerabilities within particular dimensions of aid. Although aggregate analyses are useful for understanding Chinese aid allocation practices and vulnerabilities more broadly, they may mask important details about the dynamics of Chinese aid provision[16,17]. Thus, it seems particularly likely that sector-specific research into political preferencing may reveal stark differences between aid sectors across the Chinese aid architecture.

This research will test the hypothesis that the disproportionate aid allocation to the birth regions of the current African political leaders that Dreher et al. [11] found applies to some sectors more than others. This may be due to the nature of a given sector and whether it is more susceptible to pressure to be effective and/or to other forms of oversight. This research builds on the existing literature in two distinct ways. First, disaggregating total aid into sectors of aid make it possible to identify distinctive vulnerabilities among particular sectors[18]. Second, sector level aid flows are challenging to model because the data is highly skewed data with substantial amount of zero values. In this research, we extend the research by modeling the sector level outcomes with a two-part model, instead of a single-equation model.

## 2. Conceptual framework

In this research, the Theory of Change was used to examine the links between the determinants of Chinese aid allocation, the placement of Chinese aid projects, and the role of political preferencing [19]. This theory is an outcomes-based approach that describes a sequence of events that is expected to lead to a particular outcome. It is increasingly being used in international development by governmental, bilateral, and multilateral development agencies, civil society organizations, international non-governmental organizations, and research programs intended to support development outcomes [20]. The Theory of Change is flexible and may be visually represented in different ways, but the basic elements include (i) the context for the aid intervention, (ii) long-term change that the aid intervention seeks to support, (iii) the sequence of change, and (iv) assumptions about how these changes might happen [20]. In this study, we assume that donor aid is used to procure and distribute development interventions, which must be properly deployed to produce desired outcomes. A disconnect between interventions and desired aid outcomes may result from breakdowns where aid flows are determined. To better understand where such disconnects may occur in the allocation process, this study assesses indicators relevant to three key determinants of bilateral aid allocation: recipient government behavior, donor interests, and recipient need. The literature provides very little comprehensive evidence on the more nuanced determinants of Chinese aid allocation at sector levels. However, it does contribute to the conceptual model linking aid determinants and aid flows. Within our conceptual framework we expect that aid in the social and humanitarian sectors (education, health, government, emergency, multisector and social) to be linked with the development of human resources and therefore be related to recipient needs. Whereas, aid to the economic and production sectors (communication, transportation, agriculture, and energy) should reflect Chinese economic interests. The key factors assessed within these three determinants of aid allocation and rationale for its selection are outlined in the subsequent section.

### 2.1 Political preferencing/government

Importantly for this paper, political preferencing is also a determinant of aid flows and may result in inefficient aid allocation, which would subsequently alter aid interventions, outcomes, and impact [11]. Further, some aid sectors may be more vulnerable to political preferencing than others, creating differential adverse impacts across sectors downstream. To this point, we follow Rajan and Subramanian, and instead of examining aid either in the aggregate or in a more narrow framework, we take a comprehensive perspective and examine aid according to “the use to which aid is put” by broad sectors[21]. In addition, literature has demonstrated that countries that democratize receive more aid [22]. Following the standard in the literature on democratization, Polity IV is used as an empirical measure of recipient levels of democracy.

### 2.2 Donor interests

In the conceptual model, economic resources (e.g., mines, oil/gas, ports) are included to control for the claim that Chinese aid is driven by an interest in accessing natural resources[11,23]. Chinese financing to Africa has been linked to the controversial “Angola Model” where China provides low interest loans to countries that rely on commodities, such as oil, minerals, and gas [24]. From 2004 to 2011, China made these deals to at least seven resource-rich African countries totaling USD$14 billion [24]. In addition to directly controlling for national resources, ports were included to control for China’s interest in facilitating the import and export of goods to support what has been termed as China’s “Maritime Silk Road” [25]. In 2006, trade between Africa and China totaled more than $50 billion, with oil imports from Angola and Sudan, timber from Central Africa, and copper from Zambia [26].

### 2.3 Recipient need

Measures of recipient need include geographic area of the country, population size, and road density. Nighttime light intensity is also included as a proxy for economic activity at the subnational level. Geographic area of the country as an indicator of recipient need is a key element in the aid allocation process for bilateral donors, such as China [12]. In addition, the number of people living in an area has been demonstrated to introduce systematic bias in the allocation of aid. In general, less populous countries receive more per capita aid than more populous ones [22,27,28]. A wide range of explanations have been offered for this negative relationship, including decreasing marginal benefit as population increases, limited capacity of large countries to absorb an additional amount of aid, and the potential of greater aid effectiveness in small countries [28]. Capital cities were included to control for the claim that a disproportionate amount of aid goes to capital cities. This could be politically motivated, meaning that unrest in capital cities is uniquely dangerous to recipient governments headquartered in the capital [29–31]. Other research finds aid is disproportionately allocated to the capital, not because that is where the people are but because it is where the wealthiest people are located, and these people are the target of resource transfers such as aid [30]. Next, the density of roads is included as a measure of recipient need as the length of road in a region proxies for infrastructure and capacity to support aid interventions [11]. Finally, nighttime light intensity is used extensively in the literature as a proxy for economic activity when subnational GDP is not available [32–34].

## 3. Methods

Our empirical analysis follows closely the large-scale analysis in Dreher et al[11], who study whether foreign aid from China is prone to political capture in aid-receiving countries. Our method differs from Dreher in several ways. We are not comparing the subnational allocation of Chinese and World Bank development finance. Instead, we focus explicitly on Chinese aid flows to explore whether or not this favoritism exists when aid flows are further disaggregated into sectors. Second, we use an expanded analysis sample to include aid flows and political leader data through 2014. Third, given the lack of literature on sector level Chinese aid allocation we exclude the role of elections in this analysis and focus on a more general set of covariates. S1 Table provides a detailed comparison of our variables to the variables used by Dreher et al.[11]. Finally, sector level aid flows are challenging to model because the data is highly skewed data with substantial amount of zero values. We explicitly model the zero aid outcome by a two-part model approach. The regression method we selected is a meaningful alternative and provides policy-relevant estimates.

This analysis draws data from ten secondary data sources. Sources used include data collected by government agencies, think tanks, and academic centers. Table 1 indicates the variables that were used from each data source.

**Table 1:** Analysis variables.

VARIABLE	DEFINITION	SOURCE
**Dependent Variables**		
**All Aid**		
All Aid_ict_	All official financing activities coded as Official Development Assistance and Other Official Flows	AidData. 2017. Global Chinese Official Finance Dataset, Version 1.0
**Sector Specific Aid**		
Health_ict,_ Communications_ict,_ Education_ict,_ Transportation_ict,_ Agriculture_ict,_ Emergency_ict,_ Energy_ict,_ Government_ict,_ Social_ict_	All official financing activities coded as Official Development Assistance and Other Official Flows	AidData. 2017. Global Chinese Official Finance Dataset, Version 1.0
**Time Varying Controls**		
Birthregion_ict_	= 1 if the political leader of country c in year t was born in administrative region *i*, 0 otherwise	Dreher A, Fuchs A, Hodler R, Parks B, Raschky P, Tierney M. Aid on Demand: African Leaders and the Geography of China’s Foreign Assistance. AidData Working Papers. October 2016. Archgios. A Data Set on Leaders 1875–2015 Version 4.1
**Time Invariant Controls**		
Light_ic_	Log of the average nighttime light intensity of the pixels in region *i* of country c in 2000 (i.e. beginning of sample period)	National Oceanic and Atmospheric Administration (NOAA); Version 4 DMSP-OLS Nighttime Lights Time Series
Area_ic_	Square kilometers of subnational regions	Directly calculated from the shapefile of subnational boundaries
Population_ic_	Sum of the adjusted population count of the pixels in region *i* of country *c*.	Center for International Earth Science Information Network (CIESIN); Columbia University.
Capital_ic_	= 1 if the capital city of country *c* is located in region *i*, 0 otherwise	Natural Earth; Populated Places version 3.0.0
RoadDensity _ic_	Total length of roads per square kilometer	From the shapefile of subnational boundaries (Area) and Center for International Earth Science Information Network (CIESIN); Columbia University; Global Roads Open Access Data Set, Version 1 (gROADSv1)
Mines_ic_	Log of the sum of mineral facilities in each subnational region *i* of country *c*	United States Geological Survey (USGS); Mineral Resource Data System; Mineral facilities of Africa and the Middle East (2006)
Oil Gas_ic_	= 1 if parts of an oil or gas field overlap with the area of subnational region *i* of country *c*, 0 otherwise	Lujala, Päivi; Jan Ketil Rød & Nadia Thieme, 2007. Fighting over Oil: Introducing A New Dataset‘, *Conflict Management and Peace Science* 24[3], 239–256.
Port_ic_	= 1 if a port is located in region *i* of country *c*, 0 otherwise	National Geospatial Intelligence Agency; World Port Index
Polity_c_	= 1 country is a autocracy, = 2 if country is a anocracy, = 3 if country is a democracy in country *c*, 0 otherwise	Center for Systemic Peace, Polity IV

### 3.1 Chinese aid to Africa

AidData’s Chinese Official Finance Dataset 2000–2014, version 1, provided georeferenced project-level data on Chinese aid to Africa [6]. The aid data set was constructed using open-source methodology, called Tracking Underreported Financial Flows (TUFF), designed to collect project-level data from a variety of sources from suppliers of official finance who do not participate in global reporting systems such as China, India, and Saudi Arabia [35]. This data set codes all Chinese official financing activities in adherence with OECD definitions. This data set codes all Chinese official financing activities in adherence with OECD definitions: Official Development Assistance (ODA)-like, Other Official Flows (OOF)-like, or Vague (Official Finance). Projects coded as Vague Official Finance had insufficient information to confidently classify as ODA-like or OOF-like, and were therefore excluded from the analysis as is consistent practice with other academic works. Vague Official Finance had insufficient information to confidently classify as ODA-like or OFF-like and therefore were excluded from the analysis.[35,36]. For projects that are implemented in more than one location, AidData georeferences all locations. Our dependent variables of aid (*Aid*_ict_, *Health*_*ict*,_ *Communications*_*ict*,_ *Education*_*ict*,_ *Transportation*_*ict*,_*Agriculture*_*ict*,_ *Emergency*_*ict*,_ *Energy*_*ict*,_ *Government*_*ict*,_ *Social*_*ict*_) measure Chinese aid commitments to region *i* in country *c* and year *t* in constant 2014 US dollars. If we know there are Chinese projects in a particular subnational locality, but we have no information on their monetary amounts we set the dependent variables to 0. This is a slightly stricter definition of aid than [11]Dreher et al, because we only include in our empirical approach projects that have both location and monetary information.

### 3.2 Birth region

The main variable of interest is a binary indicator *Birthregion*_*ict*,_ which indicates the birth region of the current political leader. Political leader is defined using the definition of countries’ effective leaders from Goemans et al.’s Archigos dataset, which is a database of political leaders from 1875–2015[37]. This data was updated to assign ADM1 regions to the birthplaces of political leaders of African countries.

### 3.3 Covariates

Time varying and time invariant controls, outlined in Table 1, follow Dreher et al [11]. Nighttime light intensity in the year 2000 (*Light*_*ic*_) is included as a proxy for economic activity, which is often used when official data on gross domestic project are unavailable or when official statistics might be prone to measurement error or manipulation [38,39]. Following Michalopoulos and Papaioann, Hodler and Raschky and Dreher et al. we added 0.01 to the average nighttime light intensity before taking its logarithm. This ensures that I did not lose observations with a reported nighttime light intensity of zero.[11,40,41] To control for geographical and human-population size of subnational regions the variables *Area*_*ic*_ and year 2000 *Population*_*ic*_ were included [42,43]. Population was obtained from a Center for International Earth Science Information Network dataset (GPw4) that provides globally consistent population data at a disaggregated level [42]. In this research population was used to calculate a consistent population measure at the regional level. The location of the capital city was added to account for the role of the country’s capital in aid allocation [44]. To control for the idea that China’s interest in access to natural resources drives its aid allocation, we included measures for facilities in mines, oil and gas production, and ports (*Mines*_*ic*_, *OilGas*_*ic*_ and *Ports*_*ic*_) [45–47]. The mineral facilities include mines, plants, mills, or refineries of aluminum, cement, coal, copper, diamond, gold, iron and steel, nickel, platinum-group metals, salt, and silver. We added one before taking the log. *RoadDensity*_*ic*_ was included to proxy for the ease of project implementation based on the transportation infrastructure of the region. It was directly calculated by dividing total length (km) of roads by square kilometers of subnational regions [43,48]. *Polity*_*c*_ controls for the form of country government: autocracies (characterized by authoritarian rule), anocracies (neither full democracies nor full autocracies), or democracies (institutionalized procedures for open and competitive political participation) [49]. We added binary indicators (*Before*_*ict*_*/After*_*ict*_) to control for the two years before and two years after a region is the birth region of the political leader. Country-fixed effects control for unobserved time-invariant country characteristics that may be associated with aid placement. Year-fixed effects control for annual variation in aid flows that are not attributed to other explanatory variables.

### 3.4 Region selection

Africa was selected for this analysis for several reasons. First, political conflict across Africa is often linked to pervasive use of patronage in retaining control. Leaders across the region hold onto offices by purchasing support through the distribution of state resources [50]. Therefore understanding measures of patronage, such as aid from China, is appropriate to understand the government’s controlling influence in aid outcomes. Second, China states it spends more than half of its foreign aid in 51 African countries [51]. Finally, AidData’s database provides an opportunity to examine sector-level and project level flows to China’s largest aid recipient region. Within the existing available data, there are sufficient projects to estimate the association between Chinese aid sectors and sector-specific aid allocation to birth region of the current political leader.

### 4. Sample

This analysis covers subnational units of 44 African countries available in the AidData georeferenced dataset over the 2000–2014 period. Five small island states were excluded: Cape Verde, Comoros, São Tomé and Príncipe, Mauritius, and Seychelles. Also excluded was, and Somalia since it lacks a central government. Four additional countries (Burkina Faso, Gambia, Libya and Swaziland) were excluded, because no Chinese aid was allocated between 2000–2014. Subnational units are those at the first administrative level (ADM1) such as regions, provinces, states, or districts. There are 699 ADM1 regions across the 44 African countries in this sample that were compiled into a region year panel resulting in 10,485 observations.

AidData’s dataset uses a coding system comparable to the OECD Creditor Reporting System, which assigns a standardized code for each project that reflects the sector targeted by the project. Specifically, sector coding is done by answering the question: “which specific area of the recipient’s economic or social structure is the transfer intended to foster?”[4,52] There are 21 sectors of aid in the subnational dataset. This analysis focuses on 9 of these sectors, which account for 95% of Chinese aid projects recorded as ODA-like aid from 2000 to 2014. The sectors included in the analysis are: education (CRS Code110); health (including water, sanitation, and hygiene) (CRS Codes 120, 130, and 140); government and civil society (CRS Code 150); other social infrastructure and services (CRS Code 160); transport and storage (CRS Code 210); communications (CRS Code 220); energy generation and supply (CRS Code 230); and emergency (CRS Code 700). The remaining sectors were excluded from the analysis due to either insufficient number of project locations or unspecified sector coding in the AidData dataset (S1 Appendix). Table 2 indicates the amount of aid and the count of project locations by sector.

**Table 2:** Amount of aid and project locations by sector.

Aid Sector	Amount of Aid*	Number of Project Locations
All	41.5B	1,015
Agriculture	454.3M	99
Communication	1B	194
Education	925.6	278
Emergency	114.8M	54
Energy	15.3B	151
Government	949M	123
Health	3.8B	611
Social	1.1B	92
Transportation	17.5B	348

* $USD; M = Millions, B = Billions

The final analytic dataset (n = 10,485) is a region year panel designed to define the years a political leader is in power. In total, the dataset represents USD$41.5 in ODA or OOF-like aid and covers 1,950 project-locations for which the geographical information was geocoded at an ADM1 (province, region, or district) level or lower and monetary amounts were known. In the original data, 68% of geocoded project locations have information on their respective financial commitments.

### 5. Statistical analysis

The objective of this study is to estimate whether more Chinese aid flows to the birth region of the current political leader in African countries in some sectors relative to others. Two challenges of the data on aid flows presented themselves immediately. First, a substantial proportion of regions had zero aid flows. Second, positive aid flows were highly skewed. Because of these features, we used a two-part model [53]. The first part of the two-part model estimates the probability that a region receives an aid project (= 1 if there is an aid project and 0 if no aid project is present), specified as a probit regression (Equation 1). The second part is GLM regression model of how much aid was received, conditional on an aid project being present (Equation 2). Results from equation 1 are reported as average marginal effects (elasticities) in order to emphasize the substantive and practical significance of the findings (Table 4). We used Stata’s twopm command to estimate the two-part model.

## 6. Results

### 6.1 Summary statistics

Table 3 lists the characteristics of the sample, which covers 2000 to 2014. The average amount of total Chinese aid to Africa was US$40.9 million. About, 9.6% of regions had an aid project and 5.8% of the regions were coded as a leader’s birth region over the study period. At the sector level, the average amount of aid was highest in the transportation sector ($116m) followed by the energy and social sectors ($15.9m) and health ($8.9m). The mean nighttime light intensity was 3.8 on a scale of 1 to 63. A majority of regions (69%) were anocracies.

**Table 3:** Summary statistics (2000–2014; n = 10,485 ADM1).

VARIABLE	PERCENT OF ADM1	OBS	MEAN	SD	MIN	MAX
*Dependent Variables*						** **
Chinese aid						** **
total	9.6		$40.9m	$129m	$0	$1.3b
agriculture	0.7		$6m	$12.9m	$0	$46.8m
communication	0.8		$13.2m	$36.6m	$0	$218m
education	2		4.5m	$12.7m	$0	$92.6m
emergency	0.4		2.5m	$7.6m	$0	$48m
energy	0.9		$15.9m	$272m	$0	$1.3b
government	1		$9.1m	$17.8m	$0	$106m
health	4.1		$8.9m	$55.7m	$0	$1b
social	0.7		$15.9m	$29m	$0	$129m
transportation	1.4		$116m	$170m	$0	$1b
*Independent Variables*						** **
Birth Region	5.8	10,485	0.057	0.223	0	1
Area (square kilometers)	100	10,485	37,382	70,498	4.29	639,420
Mines (count)	38	10,485	1.2	3.48	3.67	51
Nighttime Lights (0–63 scale)	96	10,485	3.8	9.75	0	61.086
Population 2000 (count)	98	10,485	26.8m	25.8m	456,703	114m
Road Density (km of road per 100 sq km of land)	99	10,485	0.176	0.366	0	8.3
Capital Region	6.7	10,485	0.067	0.25	0	1
Oil or Gas	16	10,485	0.174	0.379	0	1
Ports	11	10,485	0.105	0.307	0	1
Polity	100	10,485	1.99	0.557	1	3
Autocracy	15.6	1,635				
Anocracy	68.9	7,230				
Democracy	15.5	1,620				

m = million and b = billion; $USD

## 6.2 Main results

We used Stata MP/16.1 to estimate OLS, probit, and GLM (two part) models to obtain parameter estimates and marginal effects for two-part aid allocation models. We first estimate the probability that a region receives an aid project and then when at least one aid project is present in a leader’s birth region, we estimated the mean amount of aid the region received. We used a multivariable gamma regression analyses with a log link. Models were adjusted for capital region, nighttime lights, population, area, ports, oil, mines, road density, polity, two year birth region leads and lags, and included year and country fixed effects. The estimated coefficients for key variables and the associated cluster-robust standard errors are shown in Table 4. Detailed specification results are shown in S2 Table and S3 Table.

**Table 4:** Results from two part model for total aid by sector, ADM1, 2000–2014^a^.

	(1)	(2)	(3)	(4)	(5)
	OLS	Probit^b^	dy/dx^c^	GLM^d^	Combined dy/dx^d^
Total Aid	.557 ***	0.260***	0.022*	0.025	$2,112,157 **
	(.234)	(0.093)	(0.008)	(0.171)	($1,107,269)
Agriculture		0.248	0.01		
		(0.260)	(0.01)		
Communication		-0.201	-0.006	-1.181	$-166,000,000
		(0.247)	(0.007)	(0.904)	($413,000,000)
Education		0.437**	.013**	0.787*	$360,420*
		(0.194)	(0.006)	(0.421)	($213,927)
Emergency		0.154	0.003		
		(0.222)	(0.004)		
Energy		0.457***	0.017***	.314	19,600,000
		(0.125)	(0.004)	(0.330)	($15,300,000)
Government		-0.024	-0.0001		
		(0.250)	(0.004)		
Health		-0.005	-0.0002	0.545	$-73418
		(0.148)	(0.005)	(0.521)	($1,274,269)
Social		0.801***	.012***		
		(0.135)	(0.002)		
Transportation		0.154	0.005	-0.195	$451,097
		(0.133)	(0.004)	(.201)	($1,315,223)

^a^ The key independent variable of interest is birth region. All models control for capital region, nighttime lights, population, area, ports, oil, mines, road density, polity, 2 year leads and lags for birth regions and include time and country fixed effects.

^b^ Coefficients from part one (probit) of the two-part model. Dependent variable is a binary variable with the value of one if one or more Chinese aid projects have been committed to an ADM1 region in a given year, and zero otherwise

^c^ Marginal effects from part one (probit of the two-part model)

^d^ Shows the coefficients from part two (GLM) of the two-part model. Standard errors in parentheses clustered at the ADM1 level. Dependent variable is aid flows (total and by sector) given an aid project is present; agriculture, emergency, government and social sectors excluded due to a small number of observations (<65). This cut point was determined based on a power analysis at the 0.90 level

^e^Shows combined marginal effects from both parts of the two-part model.

*** p<0.01

** p<0.05

* p<0.1

Standard errors in parentheses clustered at the ADM1 level

The simple OLS (column 1) of total aid is qualitatively consistent with Dreher et al. and implies a significant increase (74%) in total financial flows to ADM1 regions containing the political leaders’ birth place. Moving to the two part model, the marginal effects from the probit model (column 3) indicate that the birth regions of the current political leader are 2.2 percentage points more likely to have at least some aid, significant at the 10% level. Once the aid is disaggregated to the sector level, results continue to indicate significant political preferencing. Birth regions of the current political leader are significantly more likely than the average of all of the regions to receive education (1.3 percentage points), social infrastructure and services (1.2 percentage points), and energy aid (1.7 percentage points). Although it is important to note the social infrastructure sector is based on a small number of project locations (n = 92). No significant association was found between aid flows to the birth region of the current political leader in the agriculture, communication, education, government, health or transportation sectors. Within the education sector, the coefficients for birth region are positive in both parts and statistically significant. Both the probability of aid allocation and the amount of aid conditional on any projects increase in the birth region of the current political leader. The two part model was not fully estimated in the agriculture, emergency, government, or social sectors due to small numbers of ADM1s with an aid sector. This cut point was determined based on a power analysis conducted in Stata 16.1 powering the research at the .80 level. Aid flows to the communication, government and health sectors in both parts of the model, indicate that both the probability of aid placement and the amount of aid conditional on placement decreases in the birth region of the current political leader, although these results are not statistically significant. Next, (column 5) we show the marginal effects for the combined probit and GLM version of the two-part model. The marginal effect of the birth region of the current political leader averages about USD$2.1 million for total aid. We also controlled for the last two years before the political leader came to power and the first two years after they were out of power. These marginal effects (Table 5) are of particular interest in understanding the mechanisms of favoritism. Before a political leader is in power results indicate an increase in social aid (4.6 percentage points). Again, there are a limited number of project locations in the social sector (n = 92) and therefore these results should be interpreted in light of limited project commitments. After the political leader loses power, results indicate a significant decrease in aid flows to the education (1.1 percentage points) and government sectors (0.7 percentage points).

**Table 5:** Marginal effects from probit model for total aid and by sector, ADM1, 2000–2014. Two years before and after political power.

	(1)	(2)	(3)	(4)	(5)	(6)	(7)	(8)	(9)	(10)
VARIABLES	Total Aid	Agriculture	Communication	Education	Emergency	Energy	Government	Health	Social	Transportation
Before	0.007	0.000	0.002	0.003	0.000	0.009	-0.005	-0.001	0.046*	-0.010
	(0.02)	(0.00)	(0.02)	(0.01)	(0.00)	(0.02)	(0.00)	(0.01)	(0.02)	(0.01)
After	-0.003	0.000	0.000	-0.011***	0.006	0.010	-0.007*	0.004	0.036	0.017
	(0.02)	(0.00)	(0.00)	(0.00)	(0.02)	(0.02)	(0.00)	(0.01)	(0.02)	(0.02)
Observations	10,485	1,737	3,569	5,894	3,891	4,563	5,205	7,875	6,076	6,960
Country FE	YES	YES	YES	YES	YES	YES	YES	YES	YES	YES
Year FE	YES	YES	YES	YES	YES	YES	YES	YES	YES	YES

The key independent variable of interest is birth region. All models control for capital region, nighttime lights, population, area, ports, oil, mines, road density, polity, 2 year leads and lags for birth regions and included country and year fixed effects

Dependent variable is a binary variable with the value of one if one or more Chinese aid projects have been committed to an ADM1 region in a given year, and zero otherwise

Standard errors in parentheses, clustered at ADM1 level

*** p<0.01, ** p<0.05

* p<0.1

Results are presented as marginal effects

To be clear, we do not assume that the country level fixed effects and trends account for all remaining variation in Chinese aid allocation, only that they account for variation that may be correlated with the timing and country of the political leader. Therefore, it is not possible to have a causal interpretation for these results since it is not possible to eliminate omitted variable bias at this level of granularity. However, the country and year fixed effects do control for average differences which might be important to identify the relationship between the leaders birth regions and aid when we do not have large variation in the leaders’ birth regions. We have 9,878 region-years of data in which the birth region of the current political leader is 0 and 607 in which it is 1-in 5.8% of our data, the region-year represents a birth region of the current political leader. In terms of region (between), rather than region-years; only 12% of regions were the birth region to a current political leader at least once. Conditional on a region being the birth region of the current political leader, 48% of the regions observations have a birth leader present.

Some readers might be concerned that by avoiding fixed-effects estimation at the region-level the statistically significant results might be spurious due to omitted variable bias. Therefore, we go a step further and control for (1) country-year (rather than country, year) and (2) region fixed effects for the first part of the two part model the probit specification. Table 6 shows estimations from the more rigorous model including country-year fixed effects. When including country-year fixed effects results remain qualitatively similar. Birth regions of the current political leader are significantly more likely than the average of all of the regions to receive some aid. Once aid is disaggregated to the sector level results are again qualitatively similar in that birth regions of the current political leader are significantly more likely than average to receive education, social infrastructure and services, and energy aid.

**Table 6:** Marginal effects for total aid by sector I, ADM1, 2000–2014.

	(1)	(2)	(3)	(4)	(5)	(6)	(7)	(8)	(9)	(10)
VARIABLES	Total Aid	Agriculture	Communication	Education	Emergency	Energy	Government	Health	Social	Transportation
Birth Region	0.060***	0.062	0.013	0.096***	0.026	0.106***	0.007	0.028	0.067***	0.042
	(0.017)	(0.072)	(0.082)	(0.030)	(0.042)	(0.029)	(0.005)	(0.021)	(0.015)	(0.029)
Observations	4,648	311	361	1,006	448	743	1,028	1,678	739	1,132
Country-year FE	YES	YES	YES	YES	YES	YES	YES	YES	YES	YES

The key independent variable of interest is birth region. All models control for capital region, nighttime lights, population, area, ports, oil, mines, road density, polity, 2 year leads and lags for birth regions and included country-year fixed effects

Dependent variable is a binary variable with the value of one if one or more Chinese aid projects have been committed to an ADM1 region in a given year, and zero otherwise

Standard errors in parentheses, clustered at ADM1 level

*** p<0.01, ** p<0.05, *p<0.1

Results are presented as marginal effects

When we include region fixed effects (Table 7) we find few consistent results across models and sectors. Birth regions of the current political leader are not significantly more likely than the average of all of the regions to receive some aid. Once aid is disaggregated to the sector level results indicate that the birth regions of the current political leader are significantly less likely than average to receive communication aid and significantly more likely to receive emergency and transportation aid. As an exception, our most rigorous specification does consistently indicate significant political preferencing in the social sector. Although, this result is based on a small number of project locations (n = 92) and should be interpreted with caution.

**Table 7:** Marginal effects for total aid by sector II, ADM1, 2000–2014.

	(1)	(2)	(3)	(4)	(5)	(6)	(7)	(8)	(9)	(10)
VARIABLES	Total Aid	Agriculture	Communication	Education	Emergency	Energy	Government	Health	Social	Transportation
Birth Region	0.035	0.008	-0.088**	-0.010	0.643***	0.041	0.048	-0.078	0.121***	0.118**
	(0.033)	(0.016)	(0.040)	(0.058)	(0.056)	(0.069)	(0.083)	(0.052)	(0.044)	(0.053)
Observations	4,065	232	774	966	366	897	420	1,470	602	1,410
ADM1 FE	YES	YES	YES	YES	YES	YES	YES	YES	YES	YES
Year FE	YES	YES	YES	YES	YES	YES	YES	YES	YES	YES

The key independent variable of interest is birth region. All models control for capital region, nighttime lights, population, area, ports, oil, mines, road density, polity, 2 year leads and lags for birth regions and included region (ADM1) fixed effects

Dependent variable is a binary variable with the value of one if one or more Chinese aid projects have been committed to an ADM1 region in a given year, and zero otherwise

Standard errors in parentheses, clustered at ADM1 level

*** p<0.01

** p<0.05, * p<0.1

Results are presented as marginal effects

The small number of observations contributing to the identification with region level fixed effects is the most likely reason for inconsistency in findings. The maximum number of observations is 10,485 from 699 ADM1 regions in 44 countries. However, not all of these observations contribute to the identification in the birth region effect. In the regressions with region-fixed effects, for total aid 6,420 observations are from region and years with no variation in our dependent variable, so that coefficients are identified based on the variation from the remaining, 4,065 observations. When examining this at the sector level the reduction in observations is more drastic ranging from only 232 observations in the agriculture section to 1,470 in the health sector.

### 6.3 Specification tests

We followed Deb and Norton and systematically evaluated the four main modelling choices in two-part models[54]. First, we specified a probit model for the first part of the two-part model. As a check we also specified a logit and the results in terms of marginal effects were nearly identical indicating that there was not a substantial difference between the logit and probit. The other choices in model specifications have wider implications. As previously reported, the aid flows were highly skewed, so Box-Cox transformation to normality was used for this model. We implemented the Box-Cox transformation in two ways with and without controlling for covariates. In both cases, the estimated coefficient is close to zero corresponding to the natural log transformation. In order to determine the distribution family we used a modified Park test to examine the relationship between the mean and the variable. For our sample, we observed an estimated coefficient of 1.69 indicating the use of the gamma distribution. In summary, for choices two and three the specification tests support the use of the log link and gamma distributions. Finally, we used a Pregibon’s link test to assess the reliability of the model specification. This test was not significant at the 5% level and consequently we did not add or alter any variables in the model.

## 7. Discussion

This research investigated whether and how political preferencing of Chinese aid differs across sectors. Our method substantiates the work of Dreher[11] by providing evidence that African leaders allocate substantial additional resources to their home regions. First, this research provides evidence that political preferencing does exist within some Chinese aid sectors (social, emergency, and energy), and not among others. This sector-specific analysis provides a more nuanced picture of Chinese aid than previous analyses that determine the presence of political preference according to aggregate aid flows [11]. The sectors where political preferencing exists are also those sectors that are typically associated with limited counterfactual-based program evaluation [55,56]. The use of impact evaluation to inform programming helps, as researchers for the Center for Global Development note, to “improve the effectiveness of spending and development assistance by bringing vital knowledge into the service of policymaking and program design”[57]. The results of this paper support the need for linking impact measurement and accountability particularly within the social, emergency, and transportation sectors. Second, this research supports the need within the development community for continued transparency of Chinese aid flows at the lowest levels of disaggregation possible. Such transparency generates incentives at the planning and administrative phase as well as an opportunity to conduct due diligence to avoid preferencing [58]. Although China is not held to aid principles like OECD member countries, given its recent establishment of a unified aid agency and frequent criticism from the international community regarding its programmatic standards, such due diligence may bolster China’s reputation as a responsible donor and improve the effectiveness of its programs [59].

Other research might address the current study’s limitations. Since we observe financial values at the project level and not the project-location level, project amounts are spread equally across all project locations. This practice is consistent with AidData’s Chinese aid geocoded dataset and other spatially referenced aid flows to Africa. However, it creates an artificially homogenous allocation of project aid across locations within a country. Another modeling challenge is the large fraction of observations with zero financial values, and our two-part model can only partially address this issue; future research could be based on a stronger component of financial variables. Although a power analysis was conducted at the .80 level, there are a small amount of observations for the second part of our two-part model which could limit its ability to detect a positive effect when one is to be detected. To partially deal with this issue, we included in our model-clustered standard errors at the region level. Finally, future work can also build on the theoretical underpinnings of this work. Specifically why we would expect certain sectors to be more prone to favoritism by further disaggregating aid flows and examining them at the sector level. A more nuanced understanding of the types of projects that are preferenced is needed in order to further explain allocation motivations by sector.

It is plausible that recipients approach aid effectiveness differently within sectors that justify additional aid flows. For example, reporting requirements from other donors may lead to less preference than aid from a country such as China with limited reporting requirements [23]. Finally, this research is not causally identified, so we do not know the extent that political preferencing of Chinese aid diminishes its ultimate development impact. Despite these limitations, this research provides a valuable contribution to the literature by telling a more nuanced story of Chinese aid. This research avoids categorizing Chinese aid in general terms and instead focuses on individual variations by sector. The results of this paper support the need for continued transparency of Chinese aid flows. Specifically to support the new policy framework for measuring aid flows designed to target SDGs. Such transparency generates incentives among corrupt governments to assess costs related to compliance with donor goals across different foreign aid sectors, thereby benefiting the most vulnerable on the African continent [58].

The paper’s results are not generalizable to other donors, but given China’s growing importance in foreign aid, they have great significance. One plausible explanation for this paper’s findings is that when provided the discretion to do so, political leaders may have more flexibility to preference categories of aid that have fewer reporting requirements. For instance, project-based aid flows may be more insulated from government influence than program-based aid, because project-based aid may be more likely to answer a perceived emergency. Second, sectors that rank highly in terms of policy priorities, such as those related to the Sustainable Development Goals (SDGs), carry greater incentives to implement aid effectively than sectors such as aid to the social sector. This paper illuminates which components of that aid are subject to political capture through preferencing; future research should examine how political preferencing interacts with ultimate development impact.

## Supporting information

S1 Appendix Sectors excluded from sector level analysis. (DOCX)

S1 Table Comparison of independent and dependent variables comparison of variables used. (DOCX)

S2 Table Probit results from part one of two part model for total aid by sector, ADM1, 2000–2014. (DOCX)

S3 Table GLM results from part two of two part model for total aid by sector, ADM1, 2000–2014. (DOCX)
