# Supplementary material for: Pulling the purse strings: Are there sectoral differences in political preferencing of Chinese aid to Africa?
Source: PLoS One. 2020 Apr 22;15(4):e0232126. doi: 10.1371/journal.pone.0232126 (PMC7176131; doi:10.1371/journal.pone.0232126)
Supplement: S1 Appendix — (DOCX) [file pone.0232126.s001.docx]

**S1 Appendix. Sectors excluded from sector level analysis**

Sectors excluded from the sector level analysis include: Action Relating to Debt (n=7 project locations), Business and Other Services (n=2), Developmental Food Aid/Food Security (n=10), General Environmental Protection (n=3), Industry/Mining/Construction (n=27), Non-food Commodity Assistance (n=3) Other Multi-sector (n=17), Support to Non-Governmental Organizations (n=4), Trade and Tourism (n=2), Women in Development (n=8). Also excluded was aid that was unallocated/unspecified (n=1)
