# Supplementary material for: Pulling the purse strings: Are there sectoral differences in political preferencing of Chinese aid to Africa?
Source: PLoS One. 2020 Apr 22;15(4):e0232126. doi: 10.1371/journal.pone.0232126 (PMC7176131; doi:10.1371/journal.pone.0232126)
Supplement: S1 Table — (DOCX) [file pone.0232126.s002.docx]

**Table S1. Comparison of Independent and Dependent Variables**

**Comparison of Variables Used**

| **Category of Variable** *named by Dolan & McDade* | **Variable** | **Used in Dreher et al. (2019)**(1) | **Used in Dolan & McDade** |
| --- | --- | --- | --- |
| All Aid | Chinese total flows (ODA and OOF like) *Dreher et al.*  AllAid *Dolan & McDade* | X | X |
|  | Chinese ODA-like flows (in levels) | X |  |
|  | Chinese project dummy | X |  |
|  | World Bank total flows (in levels) | X |  |
|  | World Bank IDA flows (in levels) | X |  |
| Sector Specific Aid | Agriculture |  | X |
|  | Communication |  | X |
|  | Education |  | X |
|  | Emergency |  | X |
|  | Energy |  | X |
|  | Government |  | X |
|  | Health |  | X |
|  | Social |  | X |
|  | Transportation |  | X |
| Time Varying Controls | Birthregion | X | X |
|  | Prebirth (2 years) | X | X |
|  | Postbirth (2 years) | X | X |
|  | Birthregion Spouse | X |  |
| Time Invariant Controls | Light2000 (in levels) *Dreher et al.*  Light *Dolan & McDade* | X | X |
|  | Population2000 (in levels) *Dreher et al.*  Population *Dolan & McDade* | X | X |
|  | Capitalregion *Dreher et al.*  Capital *Dolan & McDade* | X | X |
|  | Mines (in levels) | X | X |
|  | Oilgas | X | X |
|  | Area (in levels) | X | X |
|  | Ports | X | X |
|  | Roaddensity | X | X |
|  | Polity |  | X |
|  | Executive election next year | X |  |
|  | Legislative electoral competitiveness | X |  |
|  | Provincial elections | X |  |

**Variables that Appear Uniquely in Dolan & McDade**

- Sector specific aid
- Polity

**Explanation of Key Variables Found Uniquely in Dolan & McDade**

| **Variable** | **Definition** | **Data Source** |
| --- | --- | --- |
| Health, Communications, Education, Transportation, Emergency, Energy, Government, Social | All official financing activities coded as ODA and ODF like | AidData. 2017. Global Chinese Official Finance Dataset, Version 1.0 |
| Polity | =1 if country is autocracy,  =2 if country is anocracy,  =3 if country is a democracy  in country c, 0 otherwise | Center for Systemic Peace, Polity IV |

**Taken from Dolan &McDade*

**Important Clarification**

Understanding which variable from the Dreher et al. study is represented by the *AllAid_ict_* variable in Dolan & McDade is an important point of clarification for distinguishing between the data sets used in each of the studies.

Below is an excerpt from p. 49 of Dreher et al. that discusses their variable, *Aid_ict_*:

Our dependent variable, *Aid_ict_*, measures Chinese aid allocated to region *I* in country *c* and year *t* in constant 2009 US$. But we distinguish between two definitions of *Aid_ict_*. First, we analyze the allocation of Chinese “aid” in the broadest sense, including **all official financing activities from China** as identified by Strange et al. (2017). To the maximum extent possible, Strange et al. (2017) code Chinese Government-financed projects as either **“ODA-like” or “OOF-like”** based upon the OECD criteria for Official Development Assistance (ODA) and Other Official Flows (OOF)… Second, we restrict our analysis to Chinese “aid” in the strict sense of the term, i.e., those flows that Strange et al. (2017) identify as being “ODA-like.”

Given that the “definition” of the variable *AllAid_ict_* in Dolan & McDade states, “All official financing activities coded as Official Development Assistance and Other Official Flows,” The *AllAid* variable relates most closely to the Dreher et al. variable *Chinese total flows,* since the values of ODA and OOF both factor into the variable.

(1) Dreher A, Fuchs A, Hodler R, Parks BC, Raschky PA, Tierney MJ. African leaders and the geography of China's foreign assistance. Journal of Development Economics 2019.
