# Supplementary material for: Pulling the purse strings: Are there sectoral differences in political preferencing of Chinese aid to Africa?
Source: PLoS One. 2020 Apr 22;15(4):e0232126. doi: 10.1371/journal.pone.0232126 (PMC7176131; doi:10.1371/journal.pone.0232126)
Supplement: S3 Table — (DOCX) [file pone.0232126.s004.docx]

| Table S3: GLM results from part two of two part model for total aid by sector, ADM1, 2000-2014 | | | | | | |
| --- | --- | --- | --- | --- | --- | --- |
|  | (1) | (2) | (3) | (4) | (5) | (6) |
|  | Total Aid | Communication | Education | Energy | Health | Transportation |
| Birth region | 0.025 | -1.181 | 0.787* | .314 | 0.545 | -.195 |
|  | (0.171) | (0.904) | (0.421) | (0.330) | (0.521) | (.201) |
| Before | 0.813 | -1.281*** | 0.070 | -1.284** | 3.899 *** | -.829 |
|  | (0.479) | (0.186) | (0.710) | (0.690) | (1.441) | (.825) |
| After | 1.250 | 0 | -1.480* | 4.404*** | 0.321 | .173 |
|  | (1.141) | 0 | (0.765) | (1.372) | (0.596) | (.350) |
| Capital region | 0.558** | -1.829* | 2.228*** | -0.534 | -0.059 | .309 |
|  | (0.275) | (1.020) | (0.621) | (.611) | (0.486) | (.496) |
| Nighttime Lights (log) | 0.007 | 0.288*** | -0.520*** | 0'.193 | -0.004 | -.005 |
|  | (0.075) | (0.123) | (0.171) | (0.160) | (0.091) | (.178) |
| Population | -0.007** | 0'.005 | -0.003 | -.007 | -0.016 | .001 |
|  | (.003) | (0.004) | (0.005) | (0.005) | (0.011) | (.002) |
| Area | 4.44** | 8.310** | -6.298 | 4.526** | 2.833 | 2.864 |
|  | (2.35) | (4.340) | (6.453) | (2.156) | (5.698) | (3.138) |
| Ports | 0.703** | 0.394 | 0.520 | .994** | 0.009 | .014 |
|  | (.324) | (0.464) | (.449) | (0.431) | (0.735) | (.530) |
| Oil/Gas | 0.267 | 1.547 | 0.351 | -0.278 | -.0.456 | -.482 |
|  | (0.270) | (1.231) | (-0.417) | (.245) | (0.786) | (.317) |
| Mines (log) | 0.226** | 0.059 | 0.463*** | -0.154 | -0.052 | .007 |
|  | (0.107) | (0.139) | (0.194) | (.160) | (0.171) | (.127) |
| Road density | -0.054 | -3.066 | 0.129 | -0.532 | 0.066 | -.023 |
|  | (0.100) | (2.412) | (0.661) | (.774) | (0.477) | (.099) |
| Polity |  |  |  |  |  |  |
| Anocracy | -1.119** | 4.667*** | -1.238 | 0.530 | 1.800 | .199*** |
|  | (0.502) | (1.587) | (2.296) | (.609) | (2.457) | (-1.678) |
| Democracy | -3.965*** | -4.911*** | -1.683 | -2.889*** | .387 | 0 |
|  | (0.496) | (.412) | (2.477) | (.673) | (3.373) | 0 |
| Observations | 604 | 65 | 95 | 86 | 147 | 123 |
| Country FE | YES | YES | YES | YES | YES | YES |
| Year FE | YES | YES | YES | YES | YES | YES |
| Dependent variable is aid flows (total and by sector) given an aid project is present; agriculture, emergency, government and social sectors excluded due to small counts (<65). This cut point was determined based on a power analysis at the 0.80 level | | | | | | |
| Standard errors in parentheses, clustered at the ADM1 level | | | |  |  |  |
| *** p<0.01, ** p<0.05, * p<0.1 | | | |  |  |  |
| Constant includes autocracy |  |  |  |  |  |  |
| Results presented as coefficients |  |  |  |  |  |  |
