# Supplementary material for: Pulling the purse strings: Are there sectoral differences in political preferencing of Chinese aid to Africa?
Source: PLoS One. 2020 Apr 22;15(4):e0232126. doi: 10.1371/journal.pone.0232126 (PMC7176131; doi:10.1371/journal.pone.0232126)
Supplement: Supplementary file 4 [file pone.0232126.s003.docx]

Table S2: Probit results from part one of two part model for total aid by sector, ADM1, 2000-2014

	(1)	(2)	(3)	(4)	(5)	(6)	(7)	(8)	(9)	(10)
VARIABLES	Total Aid	Agriculture	Communication	Education	Emergency	Energy	Government	Health	Social	Transportation
Birth region	0.260***	0.248	-0.201	0.437**	0.154	0.457***	-0.024	-0.005	0.801***	0.154
	(0.093)	(0.260)	(0.247)	(0.194)	(0.222)	(0.125)	(0.250)	(0.148)	(0.135)	(0.133)
Before	0.079	0	0.069	0.087	0	0.191	-0.392	-0.037	1.165***	-0.381
	(0.182)	0	(0.434)	(0.399)	0	(0.435)	(0.293)	(0.273)	(0.290)	(0.333)
After	-0.031	0	0	-0.537***	0.227	0.220	-0.580*	0.097	1.010***	0.347
	(0.196)	0	0	(0.156)	(0.516)	(0.442)	(0.319)	(0.191)	(0.321)	(0.274)
Capital region	0.951***	0.391	0.901***	0.780***	0.309	0.424**	1.453***	0.672***	1.109***	0.576***
	(0.093)	(0.309)	(0.274)	(0.177)	(0.255)	(0.166)	(0.196)	(0.159)	(0.149)	(0.135)
Nighttime Lights	0.123***	0.161***	0.004	0.059	0.044	0.044	0.275***	0.145***	0.134***	0.111***
	(0.021)	(0.054)	(0.044)	(0.037)	(0.049)	(0.035)	(0.093)	(0.033)	(0.034)	(0.028)
Population	-0.002	-0.009*	-0.000	0.003	-0.006*	-0.002	-0.009	-0.002	0.006	-0.001
	(0.001)	(0.005)	(0.003)	(0.003)	(0.003)	(0.002)	(0.009)	(0.002)	(0.004)	(0.002)
Area	1.502**	3.757**	6.895***	1.333	1.091	1.829**	-5.956	0.373	1.125	0.758
	(0.622)	(1.821)	(1.953)	(0.999)	(1.200)	(0.817)	(7.493)	(0.739)	(0.947)	(0.699)
Ports	0.035	-0.930*	0.018	0.263*	0.390	0.061	0.261	-0.235	0.048	0.098
	(0.082)	(0.537)	(0.195)	(0.149)	(0.261)	(0.151)	(0.211)	(0.160)	(0.145)	(0.136)
Oil/Gas	-0.085	0.046	0.471	-0.457**	-0.113	0.263**	0.006	-0.064	-0.015	-0.034
	(0.084)	(0.322)	(0.381)	(0.211)	(0.239)	(0.134)	(0.263)	(0.145)	(0.242)	(0.124)
Mines (log)	0.065	0.055	-0.165**	0.045	-0.119	0.055	0.207	0.018	-0.025	0.139**
	(0.046)	(0.118)	(0.069)	(0.088)	(0.135)	(0.061)	(0.165)	(0.055)	(0.086)	(0.060)
Road density	-0.038	-0.184**	1.139***	0.079	0.940***	-0.406	0.783	-0.225***	0.078**	-0.028
	(0.029)	(0.088)	(0.308)	(0.051)	(0.330)	(0.315)	(1.136)	(0.068)	(0.039)	(0.036)
Polity	-2.297***	0.374	0.852***	0.533	-0.824	-0.145	0.394	0.483*	-0.513*	-0.962**
Anocracy	(0.268)	(0.453)	(0.248)	(0.360)	(0.541)	(0.355)	(0.399)	(0.264)	(0.277)	(0.469)
	-2.320***	-0.430	-0.321	0.684**	-1.455***	-0.779**	1.016***	-0.224	0	-0.044
Democracy	(0.406)	(0.726)	(0.319)	(0.278)	(0.392)	(0.315)	(0.314)	(0.338)	0	(0.216)
Constant	-1.816***	-2.437***	-3.276***	-3.434***	-1.347***	-1.786***	-3.941***	-2.130***	-3.877***	-2.503***
	(0.195)	(0.441)	(0.391)	(0.368)	(0.257)	(0.232)	(0.497)	(0.286)	(0.453)	(0.309)
Observations	10,485	1,737	3,569	5,894	3,891	4,563	5,205	7,875	6,076	6,960
Country FE	YES	YES	YES	YES	YES	YES	YES	YES	YES	YES
Year FE	YES	YES	YES	YES	YES	YES	YES	YES	YES	YES

Dependent variable is a binary variable with the value of one if one or more Chinese aid projects have been committed to an ADM1 region in a given year, and zero otherwise

Standard errors in parentheses, clustered at ADM1 level

*** p<0.01, ** p<0.05, * p<0.1

Results are presented as coefficients; pseudo R-squareds cannot be interpreted independently and therefore were not included in this table

Constant includes autocracy
